# Description of the Thermotropic Behavior Of Membrane Bilayers In Terms of Raman Spectral Parameters: A Two-State Model

**DOI:** 10.6028/jres.092.012

**Published:** 1987-04-01

**Authors:** William H. Kirchhoff, Ira W. Levin

**Affiliations:** National Bureau of Standards Gaithersburg, MD 20899; National Institutes of Health Bethesda, MD 20205

**Keywords:** logistic function, membrane, phase transition, Raman spectroscopy, two-state model

## Abstract

An analytical expression is developed for describing the thermotropic behavior of membrane bilayers as studied by Raman spectroscopy. The expression is derived from a two-state model of the main gel to liquid crystalline phase transition in lipid bilayers. Experimental data for a variety of diacylphosphatidylcholines and their derivatives have been fit by least squares to the two-state expression to within currently achievable measurement error. Numerical techniques have been developed for placing bounds on the parameters of the two-state model in situations of sparse data in the phase transition region. By fitting the model to the measured spectroscopic data, estimates of the extent of cooperativity in the phase transition can be obtained in a systematic manner.

## Introduction

In recent years the use of Raman spectroscopy for the study of the phase transition and the order/disorder behavior of both intact and reconstituted membrane bilayers has gained dramatically in popularity through the wide range of applications amenable to spectroscopic techniques [1]^1^ Although calorimetric measurements can provide accurate information on enthalpy changes that occur during phase transitions, they provide little insight into the nature of the molecular reorganization accompanying the phase transition. Since Raman spectra are particularly sensitive to the conformational, packing, and dynamical changes involving the hydrocarbon chains, bilayer reorganizations within, for example, the hydrophobic region of the membrane, may be monitored directly as a function of temperature. These observations can be made in an aqueous medium, without either the introduction of bulky molecular probes or elaborate sample preparation. By using low laser excitation powers (15–200 mW) at the sample, external perturbations to the system are minimized. Because of the sensitivity of the spectra to conformational changes, Raman spectroscopy has also been useful in elucidating the effects of intrinsic and peripheral components such as sterols and proteins on the thermal properties of membranes. In a few systems, reconstituted, naturally-occurring membranes have been studied and have provided useful information obtainable only from spectroscopic examination [2].

Although the vibrational assignments for the spectra of lipid bilayers are complex, they are understood sufficiently well to allow discussion of membrane reorganizations in terms of the observed spectroscopic frequencies, intensities and line-widths of a variety of spectral features reflecting the headgroup, interface, and acyl chain regions of the lipid components. A particularly useful approach for presenting the thermal data has been in terms of spectral peak height intensity ratios determined from either the 2900 cm^−1^ carbon-hydrogen or 1100 cm^−1^ carbon-carbon stretching mode regions. In general, these data are primarily sensitive either to alterations in the lateral interactions or to intermolecular order of the acyl chains and intrachain *trans-gauche* conformational changes, respectively.

In this paper we propose an analytical expression, based upon a two-state model of the primary phase transition phenomenon, which is useful for systematically describing the form of the temperature profiles. We also describe an approach, based on a nonlinear, least squares analysis, for extracting relevant thermodynamic information from the temperature dependence of the observed spectra. This approach is robust in handling the range of data encountered in studies of membrane phase transitions.

## Thermodynamic Model

Within the last 10 years a number of models have appeared describing within either a thermodynamic or statistical mechanical framework various features of the main phase transition in membrane bilayers. Most of these models have been critically reviewed by Nagle [3]. An additional review by Israelachvili, et al. [4] discusses the physical principles of membrane organization and includes descriptions of membrane-protein interactions and nonbilayer lipid structures. We concern ourselves here with the simplest of these phase transition models; namely, that of thermodynamic equilibrium between noninteracting domains in either one of two states [5]. We have been guided by the philosophy that the accuracy with which a simple model can quantitatively describe the experimental data serves as a guide to assessing the required features of a more complex model, to comparing and evaluating two or more models, and to defining the accuracy requirements of additional measurements.

The derivation of the thermodynamic model is presented in Appendix A in detail in order to distinguish clearly between numbers of molecules and numbers of domains in each of the two states since the observed spectral intensities are determined by the number of molecules in each of the states while the temperature dependence of the spectral intensities is determined by the distribution of domain sizes. The mathematical development follows closely the description by Hill of the two-state model for small systems [6]. The resulting analytical expression for the temperature dependence of the Raman spectra are identical to those published by Dluhy, et al. [7] using the Zimm-Bragg theory originally developed for describing polypeptide denaturation [8]. The difference between the thermodynamic and the Zimm-Bragg developments is in the interpretation of the parameters derived from the analysis. We prefer the thermodynamic development because of the simplicity of its physical interpretation. For so long as the data are described by a simple model, it seems little more than a matter of taste as to how the parameters are to be interpreted. Only when the data can be seen to contain additional information would it be fruitful to apply a more complex model. Finally, though the derivation is applied in this paper to Raman spectroscopic data, it is generally applicable to all spectroscopic studies of such cooperative phenomena.

In the two-state model, a single bilayer is considered to consist of domains of varying numbers of lipid molecules *n* with each domain existing in one of two states labeled *A* and *B.* The distribution of domain sizes and the requirement that all molecules within a domain exist either in state *A* (the low-temperature gel state) or in state *B* (the high-temperature liquid crystalline state) determine the cooperativity exhibited by the bilayers during the phase transition between the two states. The distribution of domain sizes is assumed to be temperature independent, while the number of domains in either state is temperature dependent. Although a phase transition in the strictest sense is excluded for a distribution of domains of finite size, the discontinuous nature of the phase transition will be approximated for large values of *n*.

The temperature dependence of the intensity *Iv* of a spectral feature (Raman or otherwise) that depends upon the distribution between two states of the molecules being observed is given by eq (A32) in Appendix A:

$$I=\frac{I_{Av}}{\left(1+e^{t}\right)}+\frac{I_{Bv}}{\left(1+e^{-t}\right)}$$ (1)

The dimensionless, reduced temperature *t* is defined as *t*=(*T−T*_0_)*/D*, where *T*_0_ is the midpoint of the phase transition and the scale factor *D* is related to the enthalpy of transition per mole of lipid molecules, $\Delta \bar{H}$, and the effective domain size, *n*_eff_, by:

$$D=(RT_{0}^{2})/\Delta \bar{H}n_{\text{eff}}$$ (2)

As discussed in Appendix A, eq (1) represents an approximation to a summation over a distribution of domain sizes. To test the accuracy of this approximation, sets of hypothetical data were created using a lognormal distribution [9] of domain sizes from which Raman intensities were calculated for values of *N_A_* and *N_B_*, the numbers of molecules in states *A* and *B* respectively. The values of *N_A_* and *N_b_* were calculated for the lognormal distribution using eqs (A21) and (A24). The lognormal distribution was chosen because it excludes domains of negative size and allows for a pronounced skewing to high values of *n*. This skewing causes various measures of the center of the distribution to differ significantly and provides a sensitive test of a single-term approximation to a sum over a distribution. The data sets corresponded to distributions of varying breadth which, when fit by the method of least squares to eq (1), gave the same value of *n*_eff_.

In table 1, five measures of the center of a distribution: the mode, M, which is the most populous value of *n*, the mean value of *n*, <*n*>, the root mean square of *n*, √<*n*^2^>, the median, *n*(50%), and the value of <*n*^2^>/<*n*> are compared with the value of *n*_eff_ returned by the least squares fit for three distributions of varying spread. It can be seen that as the spread of the distribution narrows, the agreement of all of these measures improves. Even though, for the broadest distribution, the values of these measures covered more than an order of magnitude, eq (1) gave a satisfactory fit with a relative standard deviation of 2%.

**Figure 1:**
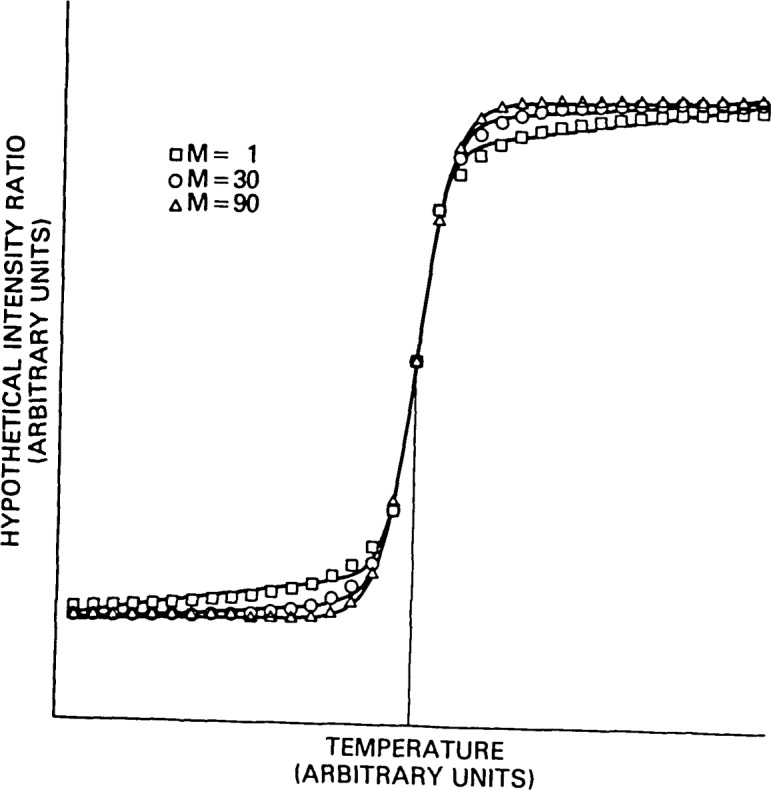
A graphical comparison of the temperature dependence of hypothetical Raman intensity ratios for three domain size distributions with modes *M* = 1, 30, and 90 and *n*_eff_=100.

Other conclusions may be drawn from the data presented in table 1. The value of *n*_eff_ obtained from the least squares fit was best approximated by the root mean square value √<*n*^2^>, and the median, *n*(50%), rather than by <*n*^2^>/<*n*> as we had initially expected. This is a function, however, of the density of data in the vicinity of the transition interval. If the data range is narrowed to include only the transition interval and if the number of data in that region is increased, then the limit of <*n*^2^>/<*n*> is approached in agreement with the observation in Appendix A that this measure gives the correct temperature dependence at the asymptotes and at the inflection point when used in a single term approximation to the sum over domain sizes. Another pronounced effect of the spread of the distribution was to give a more gradual convergence to the asymptotic limits and thus, the appearance of sloping rather than horizontal asymptotes. This can be seen in figure 1 in which the hypothetical intensity ratio profiles are drawn for each of the three distributions listed in table 1. The sloping asymptotes arise from contributions from domains of small size as originally suggested by Sturtevant [10].

A somewhat better fit to broad distributions can be obtained by modifying eq (1) in order to allow for nonhorizontal asymptotes, namely:

$$Iv=\frac{I_{Av}+I_{av}(T-T_{0})}{\left(1+e^{t}\right)}+\frac{I_{Bv}+I_{bv}(T-T_{0})}{\left(1+e^{-t}\right)}.$$ (3)

From table 1 it can be seen that inclusion of a nonhorizontal baseline in the fit of the hypothetical data improved the quality of the fit, as judged by the standard deviation, and increased the estimate of the value of *n* slightly, though not nearly enough to bring it to its expected value of <*n*^2^>/<*n*>.

**Figure 2:**
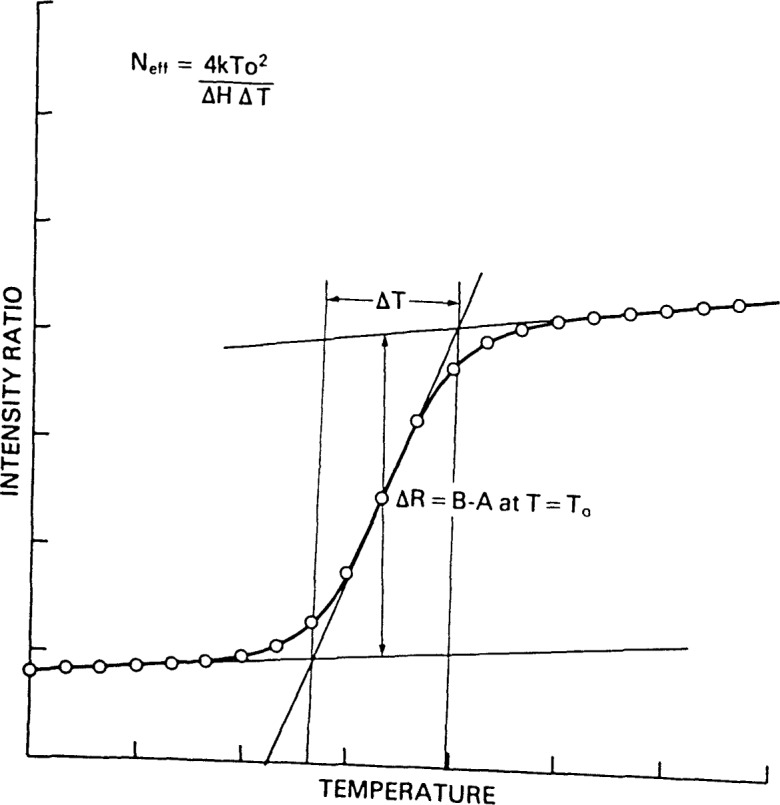
Relationship between the parameters of eq (5) and the graphical analysis of phase transition curves.

In the absence of an analytical expression such as eq (3), it has been customary to estimate *T*_0_ and the van’t Hoff enthalpies from observed temperature profiles using straight line approximations to the three distinct regions of the profiles. Thus, Δ*T* has been defined as the temperature interval between the intersection of a line drawn tangent to the phase transition curve at its inflection point and the two lines drawn tangent to the high and low temperature asymptotes. This definition of Δ*T* is illustrated in figure 2. The relationship of Δ*T* to the scale parameter *D* of eq (3) can be calculated directly to give Δ*T*=4*D*, ignoring terms of order (*I_bv_−I_av_*)/(*I_Bv_−I_Av_*). From the definition of *D* given by eq (2),

$$\Delta T=(4RT_{0}^{2})/\Delta \bar{H}n_{\text{eff}}.$$ (4)

Comparison of eq (4) with similar expressions given by Sturtevant [11] indicates that Δ*T* corresponds to the Δ*T* used for calorimetric determinations of van’t Hoff enthalpies. Care should be taken in the interpretation of *n*_eff_, however, since estimates of the slope made as close to the inflection point as possible will give values of *n*_eff_ approaching <*n*^2^>/<*n*> which, for broad distributions of domain sizes, can be an order of magnitude higher than <*n*>.

In the Raman spectroscopic experiment temperature profiles are collected over periods of several hours. Since fluctuations in the intensity of the incident laser radiation lead to corresponding fluctuations in the observed Raman intensities, the ratio of the intensities of two spectral features at different scattering frequencies provides a more precisely defined temperature profile which is relatively independent of the experimental environment. Moreover, if the vibrational features are chosen to be sensitive to the ordering of the lipid hydrocarbon chains, the temperature dependence of the intensity ratio will be more pronounced than for the individual intensities themselves.

Forming the ratio *r*_12_*=I*_1_*/I*_2_ and substituting eq (3) for *I*_1_ and *I*_2_, we obtain after rearrangement,

$$r_{12}=\frac{A+a(T-T_{0})}{\left(1+e^{t^{\prime }}\right)}+\frac{B+b(T-T_{0})}{\left(1+e^{-t^{\prime }}\right)}$$ (5)

where, to first order in *I_av_/I_Av_* or *I_bv_/I_Bv_*,

$$\begin{array}{l} A=\frac{I_{A1}}{I_{A2}},\;a=\frac{1}{I_{A2}}\left(I_{a1}-I_{a2}\left(\frac{I_{A1}}{I_{A2}}\right)\right),\;B=\frac{I_{B1}}{I_{B2}},\\ \;b=\frac{1}{I_{B2}}\left(I_{b1}-I_{b2}\left(\frac{I_{B1}}{I_{B2}}\right)\right),\;t^{\prime }=\frac{\left(T-T_{0}^{\prime }\right)}{D},\\ \;\text{and}\;T_{0}^{\prime }=T-D\ln\left(\frac{I_{B2}}{I_{A2}}\right). \end{array}$$ (6)

In the expression for $T_{0}^{\prime }$, the contributions from *I_b_*_2_ and *I_a_*_2_ have been ignored. A comparison of eqs (3) and (5) shows that the intensity ratio has approximately the same functional dependence on temperature as the individual intensities themselves, except that $T_{0}^{\prime }$, the apparent midpoint of the phase transition, has been shifted from the true value by an amount *D*ln(*I_B_*_2_/*I_A_*_2_). If, instead of *r*_12_, the temperature behavior of *r*_21_*=I*_2_*/I*_1_ is analyzed, a new apparent midpoint, $T_{0}^{\prime \prime }=T_{0}-D\ln(I_{B1}/I_{A1})$, is obtained and a comparison of the temperature dependence of *r*_21_ with *r*_12_ shows an apparent shift of the transition temperature of *D*ln(*B/A*).

Eqs (3) and (5) are the expressions we have used for the analysis of the Raman intensity profiles. Spectral data are fit by the method of least squares to Raman intensities or intensity ratios. From these fits, estimates of transition temperatures and van’t Hoff enthalpies are obtained in a systematic manner. For some systems in which this analysis has been applied, the sharpness of the phase transition requires a particularly robust least squares calculation. Therefore, the details of the least squares analysis are presented in Appendix B.

## Analysis of Temperature Profiles Derived From Raman Intensity Ratios

The analytical approach outlined in the preceding section and in the appendices to this paper have been applied to dipalmitoylphosphatidylcholine (DPPC) as well as to several cyclopentanoid analogues of DPPC [12]. In the following discussion, the results of some of these analyses will be described for the purpose of examining the limitations of the two-state model and the ways in which these limitations are made manifest by the statistical analysis.

**Figure 3:**
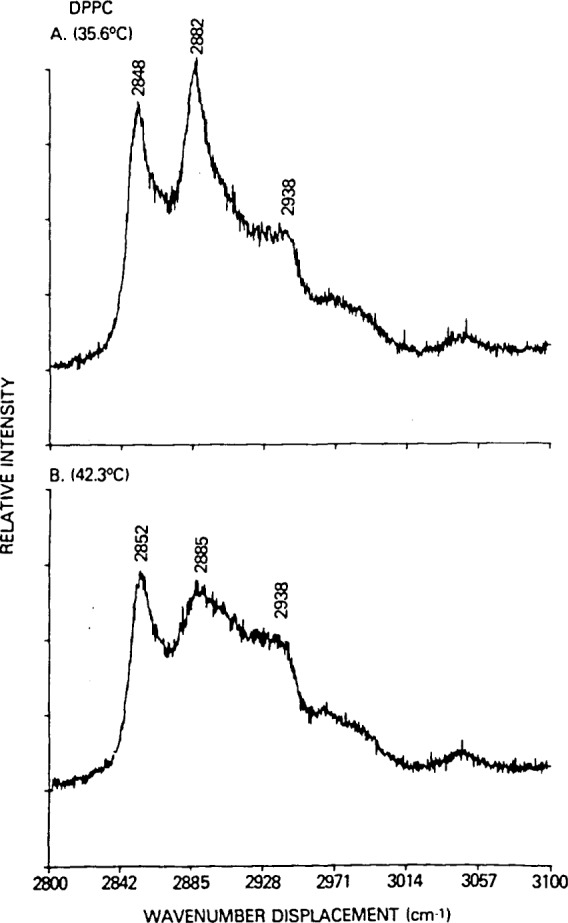
Raman spectra of dipalmitoylphosphatidylcholine in *(A*) the gel state at 35.6 °C and (*B*) the liquid crystalline state at 42.3 °C.

Although several Raman spectral regions are suitable for deriving temperature profiles from appropriate intensity ratios to assess bilayer reorganizations, we will use the 2900 cm^−1^ C-H stretching mode region for applying the present model. This congested and complex spectral interval, shown in figure 3, for the gel and liquid crystalline states of DPPC has been discussed numerous times (see, for example, ref. [1] and references contained therein). Temperature profiles can be conveniently constructed from changes in the peak height intensity ratios for the 2850, 2885 and 2940 cm^−1^ feature. These spectral transitions are assigned, respectively, to the acyl chain methylene C-H symmetric stretching modes, the methylene C-H asymmetric stretching modes and, in part, a Fermi resonance component of the chain terminal methyl symmetric C-H stretching mode [1].

**Figure 4:**
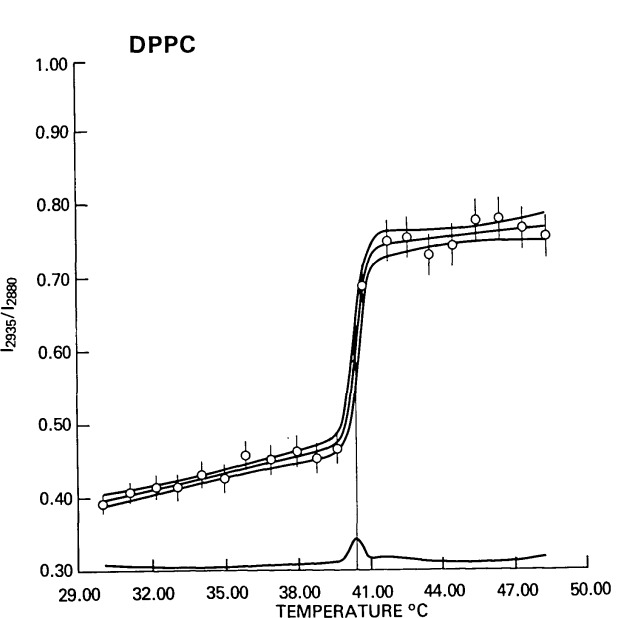
Temperature profile for the ratio of the intensity of the Raman spectral feature associated with, in part, the Fermi resonance component of the acyl chain terminal methyl symmetric C-H stretching mode at ≃2940 cm^−1^ and the methylene asymmetric C-H stretching modes at ≃2885 cm^−1^ of an aqueous dispersion dipalmitoylphosphatidylcholine (DPPC). The central curve represents the least squares fit of eq (5) to the spectral intensity data. The upper curve represents the calculated curve plus two standard deviations and the lower curve minus two standard deviations. The curve drawn along the bottom axis is the two standard deviation curve alone. The vertical bars are estimates of the uncertainties in the experimental ratios and are based on the weights of the ratios and the overall standard deviation of the fit. The values of the parameters obtained from the fit are as follows: *A* = 0.474 ± 0.006, *B* = 0.740 ± 0.012, *a* = 0.0074 ± 0.0008 and *b* = 0.0036 ± 0.0023, *T*_0_ = 40.38 ± 0.08 °C and Δ*T*<1.76 °C. The transition data range = 1.10 °C. The estimated inverse shift = −0.10 °C. The standard deviation *s*= 1.70% relative to the value of *r* at the midpoint of the transition interval.

Figure 4 illustrates the analysis of a typical phase transition profile for multilamellar dispersions of DPPC using the spectral intensity ratios constructed from the C-H stretching mode region. The values of *A, B*, *a* and *b* listed in the figure are the values of the parameters of eq (5) along with their estimated uncertainties. Only one datum, at 40.67 °C, fell within the transition interval, and consequently Δ*T* could not be determined. Illustrative of the robustness of the analysis, it was assigned a maximum value based on the temperature separation between the data on either side of 40.67 °C (39.58 and 41.78 °C) and on the standard deviation of the fit using eq (B11) in Appendix B. The transition data range given in figure 4, 1.10 K, is the value of the difference between the measured temperatures below and above *T*_0_ and should be viewed as spanning the uncertainty in *T*_0_. Ninety-five percent confidence limits for *T*_0_ can be obtained from the standard deviation of *T*_0_ returned by the least squares fit, 0.08°, by multiplying by 12.7, the value of the 95% confidence limits of a student’s *t* distribution with one degree of freedom, giving 1.02 K in agreement with the transition data range. As might be expected, the value of *T*_0_ as determined by the least squares fit is quite sensitive to the value estimated for Δ*T*. The estimated inverse shift presented in figure 4 is calculated from *D*ln(*B/A*) and is the shift in *T*_0_ which would have been obtained if *I*(2885)/*I*(2940) had been analyzed instead of *I*(2940)/*I*(2885). The vertical bars at each data point in figure 4 are twice the value of the uncertainties in the intensity ratios as estimated from the standard deviation of the fit by eq (B1). The three curves passing through the data values correspond to the profile calculated from eq (5) along with plus or minus two standard deviations. Along the horizontal axis of figure 4 is the two standard deviation curve alone. The peak in the uncertainty curve at *T*_0_ signifies that *T*_0_ is being determined almost exclusively by the value of the intensity ratio at the one datum in the transition interval relative to the separation between *A* and *B.*

**Figure 5:**
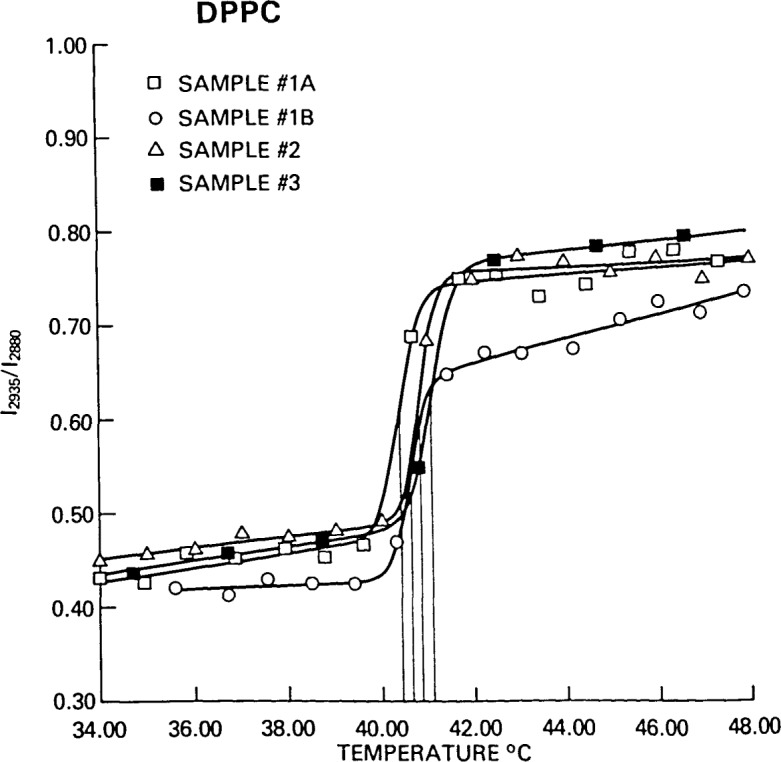
A comparison of four temperature profiles for dipalmitoylphosphatidylcholine (DPPC). The spectra were measured over a period of two years by three different investigators. Spectra for Samples No. 1 and No. 3 were recorded with a scanning spectrophotometer whereas spectra for Sample No. 2 were determined with a spectrograph. All three samples were prepared by the individual investigators using different sources for the lipid. The range in *T*_0_ was 40.4 °C to 41.1 °C.

Figure 5 illustrates several of the limitations of the simple two-state model. Four phase transition curves for DPPC from our laboratory using data from different investigators, different spectrometers, and over a two-year interval were compared. Even though the data were separated by 2° intervals, and only one, but always one, datum fell within the transition interval, the analysis provided a surprisingly reproducible phase transition temperature of 40.38 to 41.09 °C, as compared with the more accurate value of 41.55 °C measured calorimetrically by Albon and Sturtevant [5]. The maximum value of Δ*T* varied from 1.42 to 1.91 °C and reflected varying temperature data intervals and intensity measurement precision.

Using the calorimetric data for DPPC measured by Albon and Sturtevant, namely, $\Delta \bar{H}=8.50\;\text{kcal}/\text{mole}$ and *T*_0_=314.70 K, and the value obtained for Δ*T*, minimum values of the cooperative unit of 48 to 65 were calculated from eq (4). If values for the cooperative unit of 100 to 1000, which have been estimated for the phase transition in DPPC, are to be obtained from the analysis of Raman intensity profiles, measurements must be made with temperature increments of 0.1 °C in the phase transition region and with temperature control on the order of 0.01 °C.

If, for the moment, the data for the second run of sample #1B are excluded from figure 5, the values of *A, B, a* and *b* all agree to within three standard deviations as estimated by the least squares fit. For the temperature profile of sample #1B, which was measured within 24 hours of the profile of sample #1A, the values of *A*, *B, a* and *b* all varied by more than five standard deviations from the average of the other three profiles. We interpret this observation as an indication that the sample had not yet annealed into its preferred low temperature state. Its bilayer structure appeared, on comparison with the values of the gel state intensity ratios for the other samples, to be as ordered at low temperatures, although it did not exhibit the gradual increasing disorder associated with the positive value of *a.* Its phase transition temperature, though somewhat higher than the others, did not change significantly nor did the transition interval to within measurement precision. The transition concluded at a much less disordered state than the other samples and then, corresponding to a large value of *b*, continued to disorder at a much greater rate than the others until reaching a comparable intensity ratio approximately 10 °C above the phase transition temperature. Although the two-state model cannot explain the molecular origins of such behavior, the fit of the data to eq (5) provides a basis of the intercomparison of temperature profiles in a methodical, reproducible, and quantitative manner.

The values of the asymptotic slopes observed for the temperature profiles of intensity ratios cannot be interpreted on the basis of domains of small size alone. Even for the three conforming profiles in figure 5, the values of the slopes at high and low temperature are on the order of 0.5% to 1.2%, somewhat greater than the value of 0.46% obtained for the broadest domain size distribution reported in table 1. The slopes are most readily interpreted as the ability of the hydrocarbon chains of the lipid molecules to form *gauche* bonds near the acyl chain termini at the bilayer center without triggering the phase transition and, for the liquid crystalline phase, as a continuing disordering following completion of the phase transition.

**Figure 6:**
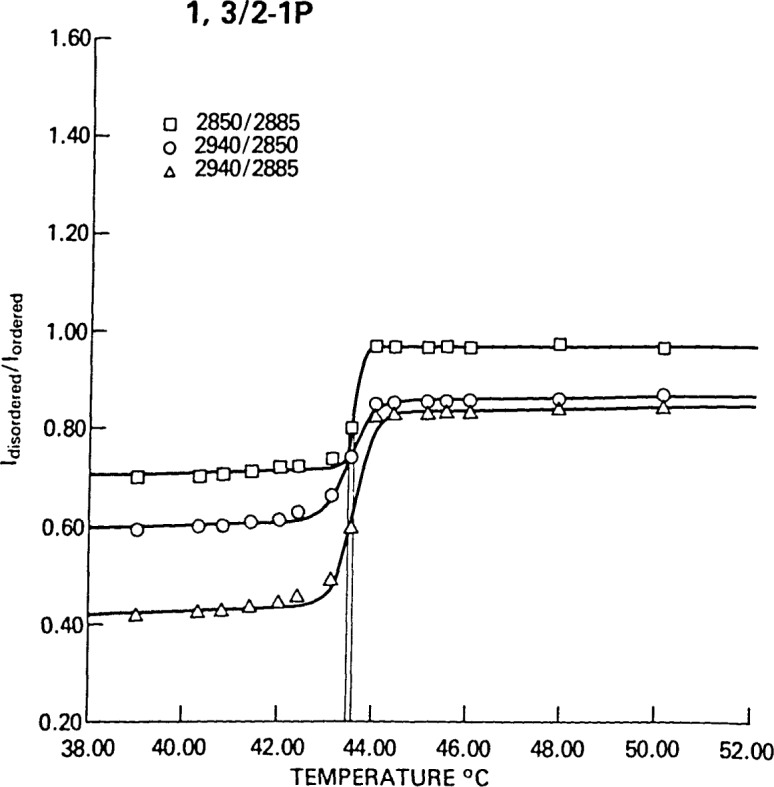
A comparison of the temperature profiles for three spectral features in the C-H stretching region for 1,3/2-1P-dipalmitoylcyclopentane-1,2,3-triol phosphatidic acid. For the 2850/2885 profile (□), *T*_0_=43.6 °C and Δ*T*=0.6 °C; for the 2940/2850 profile (○), *T*_0_ = 43.4 °C and Δ*T*=1.0 °C; for the 2940/2885 profile (△), *T*_0_ =43.6 °C and Δ*T*=0.8 °C.

Figure 6 compares the temperature profiles derived from the *I*(2850 cm^−1^)/*I*(2885 cm^−1^), *I*(2940 cm^−1^)/*I*(2850 cm^−1^) and *I*(2940 cm^−1^)/*I*(2885 cm^−1^) peak height intensity ratios for 1,3/2- 1P-dipalmitoylcyclopentane-1,2,3-triol phosphatidic acid, an analog of DPPC in which the glycerol backbone is replaced by a more rigid cyclopentane triol [13]. All three profiles give the same value for the phase transition temperature within the limits of the standard deviations obtained from the least squares fit. The values of Δ*T*, however, vary, and this variation can be used as a test of a structure-based model for the phase transition, since the *I*(2850 cm^−1^)/*I*(2885 cm^−1^) ratio reflects primarily lateral chain-chain interactions, while the *I*(2940 cm^−1^)/*I*(2885 cm^−1^) ratio reflects *trans-gauche* conformational changes in addition to the lateral effects [14]. This suggests that different chain interactions as probed by different spectral regions may either exhibit different degrees of cooperativity in the phase transition, different energies for the transition, or a combination of both effects.

**Figure 7:**
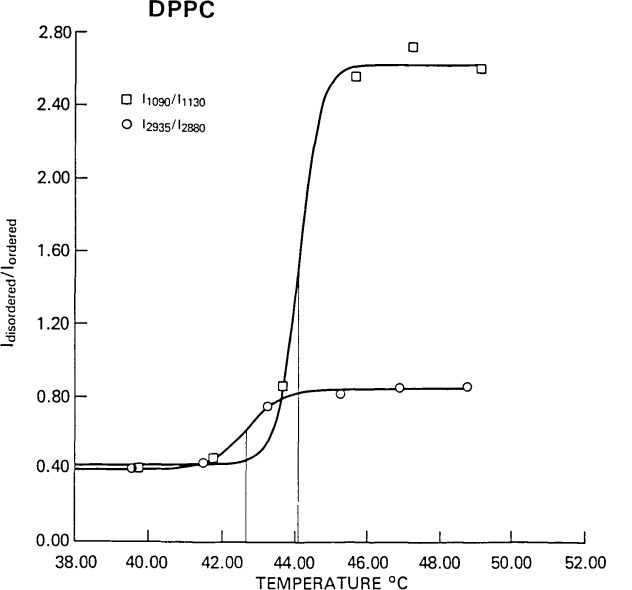
A comparison of the temperature profiles for the C-C stretching and the C-H stretching mode regions for 1,3/2-1P-dipalmitoylcyclopentane-1,2,3-triol phosphatidic acid. The difference in *T*_0_ for the two spectral regions is 1.5 °C. Spectral data for both stretching mode regions were generated simultaneously.

An even more striking comparison of temperature profiles for the cyclopentanoid derivative of DPPC can be seen in figure 7. Simultaneous measurements of the C-H stretching and the C-C stretching mode regions showed a shift of 1.5° for the transition temperature for the C-C stretching profile, a shift which cannot be explained by the choice of intensities for forming the ratio as suggested by the discussion accompanying eq (6). Although the temperature intervals were too coarse to obtain precise values of Δ*T*, the difference in *T*_0_ between the two profiles suggests that lattice expansion precedes the intrachain *trans-gauche* isomerization as the bilayer undergoes the phase transition. (The change in the intensity ratio in the C-H stretching region is not only a function of the degree of *trans-gauche* isomerization but also the interactions between neighboring hydrocarbon chains [1].) It is just this type of molecular-scale information that provides insight into the molecular mechanism of the phase transition. Also, without a statistical estimate of the uncertainty in *T*_0_, it would not be possible to assess whether the observed temperature difference is statistically significant.

Finally, returning to figure 6, an asymmetry can be observed in the shapes of profiles which cannot be explained by a thermodynamic equilibrium model. The curvature at low temperature appears to be gradual, while at high temperature the curvature is quite sharp. Such an asymmetry is suggestive of a coalescing of domains of liquid crystalline lipid to give a narrower distribution of domains of larger size toward the end of the transition. This behavior is similar to that implied by the model of Freire and Biltonen [15], and observed by Marsh et al., using electron spin resonance techniques to examine the DPPC phase transition [16].

## Conclusions

We have outlined a systematic approach for extracting phase transition temperatures and van’t Hoff enthalpies from temperature profiles derived from Raman spectral peak height intensity ratios. Within the degree of precision of the experimental data; namely, temperature intervals of 1–2 °C, temperature measurement accuracy of 0.1 °C, and peak height intensity measurement accuracy of 1 to 5%, the simple, equilibrium, two-state model implied by eq (5) fits most of the experimental data to within measurement error. Alternatively, with the above degree of precision of the experimental data, it will not be possible to distinguish between more detailed models. As a next step, therefore, in the systematic evaluation of phase transition behavior through Raman spectroscopic studies, the precision of the temperature and intensity measurements must be improved by an order of magnitude while decreasing the temperature intervals throughout the phase transition region by an order of magnitude.

The advantage of deriving an analytical expression for the Raman intensity behavior as a function of temperature lies in the development of statistically based assessments of competing models. Data such as the exact profile curvature immediately preceding and following the phase transition and hysteresis effects can be used for model testing only when the data are reproducible and the measurement accuracy is controlled. A fruitful approach is to combine improvements in the model with improvements in the experimental procedures, while using the statistical fit of analytical expressions to the data as a basis for testing both the model and the adequacy of the data.

We appreciate the limitations of the present model. Although the two-state model conveys no information concerning the molecular nature or origin of the phase transition, the vibrational spectroscopic data provide a means for directly monitoring structural changes within the lipid bilayer over a large temperature range. The spectroscopic data are thus complementary to the calorimetric data. Moreover, the extraction of thermodynamic information from the spectroscopic studies provides confirmation that the spectroscopic and calorimetric studies are responding to the same phenomena. This aspect is particularly important in the study of lipid systems where sample history influences the manifestation of conformational changes. As the next step in the development of the model, further detail concerning boundary effects between domains should be included along with a variation of the domain size distribution through the phase transition.

With an increased number of parameters in an analytic expression for the phase transition profiles comes a demand for greater data density throughout the phase transition region. We have already indicated the need for decreased temperature intervals. The comparison of temperature profiles obtained from different spectral regions promises to provide extremely useful tests for molecular-scale descriptions of the phase transition and the attendant molecular dynamics.

## Figures and Tables

**Table 1:** Comparison of a single term fit to three distributions.

	Mode	1	30	90
Distribution Parameters	Range in *n*^a^	1–11, 679	1–3, 963	21–387
	<*n*>	36.3	79.2	98.2
	√ <*n*^2^>	113.7	109.5	101.0
	*<n*^2^>/<*n*>	356.3	151.3	104.0
	median	117	110	101

Least Squares Fit to Eq 1	*n*^eff^	100	100	100
	*s*^b^	2.1%	1.2%	0.2%

to Eq 3	*n*_eff_	136.2	113	101
	*s*	1.0%	0.8%	0.2%
	slope^c^	0.46%	0.24%	0.02%

a Range: values of *n* for which the population >10^−8^ × population of mode.

b *s*: relative (to the value of *A–B*) standard deviation of the least squares fit.

c slope: value of baseline slope relative to the value of *A–B*.

## Footnotes

1 Figures in brackets indicate literature references.

## APPENDIX A

### Development of an Analytical Expression for Describing the Effect of Temperature on the Spectrum of a Molecular System in Terms of a Two-State Model

The total Raman scattered intensity *I* at a particular frequency *v* can be expressed as:

$$I_{v}=I_{Av}N_{A}+I_{Bv}N_{B},$$ (A1)

where *I_Av_* and *I_Bv_* represent the scattering intensities per molecule of molecules in each of two states *A* and *B* respectively and *N_A_* and *N_B_* are the number of corresponding molecules per scattering volume. The presence of other substances which may contribute to the overall scattering intensity will not affect the derivation that follows, since an expression of the form

$$I_{v}=I_{Av}N_{A}+I_{Bv}N_{B}+I_{Cv}N^{\prime },$$ (A2)

where *N*′ is the number of other scattering molecules, can be rewritten as

$$\begin{array}{l} I_{v}=I_{Av}N_{A}+I_{Bv}N_{B}+I_{Cv}N^{\prime }(N_{A}+N_{B})/N\\ \;=I_{Av}^{\prime }N_{A}+I_{Bv}^{\prime }N_{B}. \end{array}$$ (A3)

$I_{Av}^{\prime }$ and $I_{Bv}^{\prime }$, as defined, are functions, therefore, of the composition of the sample as well as the intrinsic scattering properties of the molecules of interest in states *A* and *B.* The temperature dependence of *I_v_*, however, will be determined by the temperature dependence of *N_A_* and *N_B_.* The purpose of the following mathematical treatment is to determine an explicit analytical expression for this temperature dependence. And since the treatment deals only with the numbers *N_A_* and *N_B_*, it is also applicable to absorption spectra in concentration ranges where the transmitted intensity or its logarithm is a linear function of concentration; that is, where Beer’s Law is obeyed.

We let ${\hat{N}}_{n}$ be the number of domains of size *n* of which ${\hat{N}}_{nA}$ are in state *A* and ${\hat{N}}_{nB}$ are in state *B.* The number of molecules in states *A* and *B* are then

$$N_{A}=\sum_{n}n{\hat{N}}_{nA}$$ (A4a)

and

$$N_{B}=\sum_{n}n{\hat{N}}_{nB}.$$ (A4b)

In a closed, one-component system in contact with a heat bath at pressure constant pressure *p*, the differential expression for the internal energy of the system is

$$dE=TdS-pdV+\sum_{n}\mu_{n}{\hat{N}}_{n}dn+\sum_{n}{\hat{\mu }}_{n}d{\hat{N}}_{n}$$ (A5a)

where

$$\mu_{n}=(1/{\hat{N}}_{n}){\left(\partial E/\partial n\right)}_{S,V,{\hat{N}}_{n},m}$$ (A5b)

and

$${\hat{\mu }}_{n}={\left(\partial E/\partial {\hat{N}}_{n}\right)}_{S,V,n,{\hat{N}}_{m}}$$ (A5c)

and where *m* refers to domains of all sizes other than *n.* The terms $\mu_{n}{\hat{N}}_{n}dn$ and ${\hat{\mu }}_{n}d{\hat{N}}_{n}$ represent a separation of the total chemical potential of the system into two factors: one factor associated with changing the number of molecules in a given domain and the other with changing the number of domains of a particular fixed size. In considering the effects of temperature on the number of domains in state *A* or state *B*, processes involving d*n* will be excluded. That is, only processes involving $d{\hat{N}}_{nA}$ and $d{\hat{N}}_{nB}$, where $d{\hat{N}}_{nA}=-d{\hat{N}}_{nB}$, will be allowed.

The Gibbs free energy *G* for a system consisting of a mixture of domains of varying but distinguishable size in one of two states, *A* or *B*, can then be written as:

$$G=\sum_{n}{\hat{\mu }}_{nA}{\hat{N}}_{nA}+{\hat{\mu }}_{nB}{\hat{N}}_{nB}$$ (A6)

where

$$\hat{N}=\sum_{n}{\hat{N}}_{n}=\sum_{n}{\hat{N}}_{nA}+{\hat{N}}_{nB}$$ (A7)

is the total number of domains in the system and where the chemical potentials ${\hat{\mu }}_{nA}$ and ${\hat{\mu }}_{nB}$ are the free energies of domains of size *n* in states *A* or *B* respectively. The condition for equilibrium between states *A* and *B* is then:

$$\Delta G=\sum_{n}{\hat{\mu }}_{nB}-{\hat{\mu }}_{nA}=0$$ (A8)

We define the partition coefficients for the system of domains as:

$$Z=\prod_{n}Z_{nA}Z_{nB}$$ (A9)

where

$$Z_{ni}=\frac{\xi_{ni}^{\hat{N}i}}{({\hat{N}}_{ni})!}$$ (A10)

and ζ *ni* is the partition function for a domain of size *n* in the state *i*. The chemical potential can also be defined in terms of the Gibbs free energy as:

$${\hat{\mu }}_{ni}={\left(\partial G/\partial {\hat{N}}_{ni}\right)}_{T,p,n,{\hat{N}}_{mj}}$$ (A11)

and the Gibbs free energy can be expressed in terms of the partition function *Z* as:

G=−kTlnZ+pV. (A12)

Combining equations (A8) through (A12) and invoking Stirling’s approximation for the logarithm of factorials gives:

$$\frac{{\hat{N}}_{nA}}{{\hat{N}}_{nB}}=e^{-\Delta G_{n}^{0}/kT}$$ (A13)

where

$$\Delta G_{n}^{0}=-kT\ln\frac{\xi_{nB}}{\xi_{nA}}$$ (A14)

is the standard state free energy change for domains of size *n.* The number of molecules in state *A* is then given by:

$$N_{A}=\sum_{n}n{\hat{N}}_{nA}=\sum_{n}\frac{n{\hat{N}}_{n}}{1+e^{-\Delta G_{n}^{0}/kT}}$$ (A15a)

and in state B by:

$$N_{B}=\sum_{n}n{\hat{N}}_{nB}=\sum_{n}\frac{n{\hat{N}}_{n}}{1+e^{-\Delta G_{n}^{0}/kT}}.$$ (A15b)

The temperature dependence of the intensity of a Raman spectral feature, which depends on *N_A_* and *N_B_*, is contained in the terms $\Delta G_{n}^{0}/kT$. As defined, $\Delta G_{n}^{0}$ is the difference in free energy between two domains of size *n*, one in state *A* and one in state *B.* For the simplest approximation to this difference, we let $\Delta G_{nA}^{0}=n\Delta G_{A}^{0}$ and $\Delta G_{nB}^{0}=n\Delta G_{B}^{0}$ where $\Delta G_{A}^{0}$ and $\Delta G_{B}^{0}$ are the standard state free energies of individual molecules in states *A* and *B* respectively. This approximation is not exact because changes in the free energy of the domains will be affected by the domain boundaries. That is, we have defined the free energy of domains strictly for the internal degree of freedon associated with the order-disorder phase transition of the lipid hydrocarbon chains. The free energy changes associated with this phase transition involve not only intramolecular but intermolecular interactions as well. Individual molecules at the boundaries between domains in different states will possess different intermolecular interaction energies in comparison with molecules within the domains themselves. The factorization of the partition function *Z* in eq (A9) into a product of single domain partition functions is also one that ignores boundary effects by assuming that the energy of interaction between domains is negligible. $\Delta G_{A}^{0}$ and $\Delta G_{B}^{0}$ as defined represent an average value of the free energy per lipid molecule for an average domain size. The definition of what constitutes an individual molecular species is flexible. A molecular species could refer to a complete lipid molecule, a single hydrocarbon chain or even a single -CH_2_-CH_2_-CH_2_-triad, the smallest unit for defining a *trans* or *gauche* conformation. The important point is to ensure that the single unit free energy, $\Delta G_{A}^{0}$ or $\Delta G_{B}^{0}$, is consistent with the definition of that single molecular unit. In the remainder of this discussion, the unit will be taken as a single lipid molecule consisting of two chains.

Substitution of $\Delta G_{nA}^{0}=n\Delta G_{A}^{0}$ and $\Delta G_{nB}^{0}=n\Delta G_{B}^{0}$ into eq (A15a) yields:

$$N_{A}=\sum_{n}\frac{n{\hat{N}}_{n}}{1+e^{-n\Delta G^{0}/RT}}.$$ (A16)

Note that a molar scale has been adopted for the exponential term so that Δ*G*^0^ is the free energy change per mole of lipid molecules, *R* is the gas constant, but *n* retains its definition as the number of molecules per domain. If *n* is large and if Δ*G*^0^ differs from 0, the exponential value is eq (A16) rapidly approaches 0 or infinity depending on the sign of Δ*G*^0^ and exhibits the discontinuity characteristic of a simple, first order phase transition. For smaller values of *n* (typically <100), the system exhibits a more continuous transition from state *A* to state *B* and can be described in a manner analogous to a two-component system in chemical equilibrium. Since we know from experiment that pure lipid bilayers exhibit sharp phase transitions, we anticipate that *n* will be sufficiently large to allow expansion of Δ*G*^0^*/T* in a power series about *T=T*_0_ the temperature at which *N_A_=N_B_*:

$${\Delta G^{0}/T=(\Delta G^{0}/T)|}_{T=T_{0}}{+\partial (\Delta G^{0}/T)/\partial T|}_{T=T_{0}}(T-T_{0}){+1/2\;\partial^{2}(\Delta G^{0}/T)\partial T^{2}|}_{T=T_{0}}{\left(T-T_{0}\right)}^{2}.$$ (A17)

We define the standard state for each domain as the one for which *N_A_=N_B_*, so that Δ*G*^0^=0 at *T=T*_0_. The derivative of Δ*G*^0^*/T* with respect to *T* can be evaluated from the Gibbs-Helmholz equation expressed in terms of differences in partial molar enthalpies; namely,

$$\partial (\Delta G^{0}/T)/\partial T=-\Delta \bar{H}/T^{2}$$ (A18)

where $\Delta \bar{H}={\bar{H}}_{B}-{\bar{H}}_{A}$. From this expression the second derivative of Δ*G*^0^*/T* with respect to *T* can be calculated:

$$\partial^{2}(\Delta G^{0}/T)/\partial T^{2}=2\Delta \bar{H}/T^{3}-(1/T^{2})(\partial \Delta \bar{H}/\partial T)$$ (A19)

Identifying $\partial \bar{H}/\partial T$ as the molar specific heat at constant pressure, *C_p_*, substituting eqs (A18) and (A19) into eq (A17), and rearranging, the Taylor series expansion of Δ*G*^0^*/T* becomes:

$$\Delta G^{0}/T=-(\Delta \bar{H}/T_{0})(T-T_{0})/T_{0}+(\Delta \bar{H}/T_{0}-\Delta C_{p}/2){\left[(T-T_{0})/T_{0}\right]}^{2}$$ (A20)

In general, phase transitions for lipids have been observed to be quite sharp with complete conversion occurring over a range of less than 10 K. Thus, at phase transition temperatures on the order of 300 K, (*T−T*_0_)/*T*_0_*<.*03 for *T−T*_0_< 10 K. Unless Δ*C_p_* is an order of magnitude larger than $\Delta \bar{H}/T_{0}$, the second term of eq (A20) can be ignored.

Wilkinson and Nagle have measured the heat capacities of several lipids within 5 K above and below their main phase transitions [17]. As the phase transition temperature is approached from lower temperatures, *C_p_* increases dramatically over the increase in *C_p_* observed for normal alkanes [18]. Above *T*_0_, *C_p_* is higher for the lipid systems than for the alkanes and decreases with increasing temperature. However, Δ*C_p_* does not appear to increase significantly and may actually be lower than the value determined for the solid-liquid phase transitions in alkanes. The anomalously high values of *C_p_* for lipids can be expected to occur when the phase transition is abnormally sharp; namely, over a temperature range of a degree or two where (*T*−*T*_0_)/*T*_0_<.005. Consequently, we assume that the temperature dependence of Δ*G*^0^*/T* can be adequately described by the first term in eq (A20).

Substitution of the first term of eq (A20) into eq (A16) gives for the number of molecules in state *A*,

$$N_{A}=\sum_{n}n{\hat{N}}_{n}/(1+e^{tn})$$ (A21)

where, for simplicity, we have defined a reduced temperature *t* as

$$t=(T-T_{0})/D=\Delta \bar{H}(T-T_{0})/RT_{0}^{2}$$ (A22)

and the scale factor *D* as

$$D=RT_{0}^{2}/\Delta \bar{H}$$ (A23)

Similarly,

$$N_{B}=\sum_{n}n{\hat{N}}_{n}/(1+e^{-tn})$$ (A24)

The magnitude of *D* may be estimated by letting $\Delta \bar{H}≃10\;\text{kcal}/\text{mole}$ and *T*_0_≃330 K so that *D*≃20 K. For *n* = 100 and *T − T*_0_> 1 K, *N_A_ <N/*100.

Equations (A21) and (A24) are still too complex to be used for a least squares analysis of the temperature dependence of spectral intensities in the vicinity of a phase transition. The next step in the development is to replace the summations in eqs (A21) and (A24) with some suitable average over the distribution of domain sizes. The distribution of domain sizes is given by ${\hat{N}}_{n}$ as a function of *n*, where ${\hat{N}}_{n}$ satisfies the following conditions:

$$N=\sum_{n}n{\hat{N}}_{n}$$ (A25)

and

$$\hat{N}=\sum_{n}{\hat{N}}_{n}$$ (A26)

and where the moments of the distribution are defined as

$$<n^{k}>=(1/\hat{N})\sum_{n}{\hat{N}}_{n}n^{k}$$ (A27)

The substitution of *<n*> into eq (A21),

$$N_{A}=\hat{N}<n>/(1+e^{t<n>}),$$ (A28)

would imply that <*n*>*^k^* = <*n^k^*> for all *k*, which would be inaccurate for all but the narrowest of distributions. A better approximation is

$$N_{A}=\hat{N}<n>/(1+et^{<n^{2}>/<n>}).$$ (A29)

Equations (A28) and (A29) both give the correct asymptotic behavior when compared with eq (A21). Both expressions have inflection points at *t* = 0. However, eq (A29) also gives the correct slope at *t* = 0, namely $-\hat{N}<n^{2}>/4$, whereas eq (A28) gives $-\hat{N}<N{>}^{2}$.

Since eq (A29) is congruent with eq (A21) at *t* = 0 and ± ∞ and since neither eq (A29) nor (A21) has any minima or maxima other than + ∝ and **−** ∞, we conclude that eq (A21) may be approximated by a single term, namely

$$N_{A}=N/(1+e^{tn_{\text{eff}}})$$ (A30)

where $N≃\hat{N}<n>$ and $n_{\text{eff}}≃<n^{2}>/<n>$. Similarly, eq (A24) can be approximated by

$$N_{B}=N/(1+e^{-tn_{\text{eff}}})$$ (A31)

where *N* and *n*_eff_ have the same values as in eq (A30) in order to conserve the total number of molecules in the system. In practice, *N* and *n*_eff_ will be adjusted to give the best overall agreement between eqs (A30,A31) and (A21,A24).

The accuracy of this single-term approximation can be tested by assuming a distribution function for *n* and comparing eq (A30) directly with eq (A21). This was done for a set of lognormal distributions as described in the main body of this paper.

Equations (A30) and (A31) can now be used in eq (A1) to describe the intensity of a Raman spectral feature as a function of temperature:

$$I=\frac{I_{Av}}{\left(1+e^{t}\right)}+\frac{I_{Bv}}{\left(1+e^{-t}\right)}$$ (A32)

where, as before, *t*=(*T−T*_0_)/*D*, but where the explicit reference to *n*_eff_ has been absorbed into the definition of *D*, namely,

$$D=(RT_{0}^{2})/\Delta \bar{H}n_{\text{eff}}$$ (A33)

and where explicit reference to the number density *N* has been absorbed into the definitions of the scattering intensities *I_Av_* and *I_Bv_.*

## APPENDIX B

### The Least Squares Fit

A computer program has been written to analyze the temperature dependence of Raman spectral data by fitting the data by the method of least squares to eq (5). Several features of this program are worth noting. Equation (5) is a linear function of the parameters *A, B, a* and *b* and a nonlinear function of *T*_0_ and *D.* Thus, the parameters of Eq (5) are evaluated using an iterative approach in which *r* is expanded in a Taylor series for each of the parameters about its currently estimated value. The series is terminated at its linear terms. The values of *r*_obs_−*r*_calc_ are used as the dependent variables and the derivatives of *r* with respect to each of the parameters as the independent variables. From a linear fit of *r*_obs_−*r*_calc_ to the derivatives of *r*, the corrections to the parameters are obtained and the process is repeated using the new values of the parameters until the changes in the parameters for each iteration are significantly lower than the standard deviations of the parameters.

The least squares fit is a weighted fit. If we assume that errors in the measured intensities are random, uncorrelated and are drawn from a single, normally distributed population of errors, and if we let *s* be the estimate of the standard deviation of that population, then the variance of a particular value of an intensity ratio is given by

$$\begin{array}{l} s^{2}(r_{12})={\left(\partial r_{12}/\partial I_{1}\right)}^{2}s^{2}(I_{1})+{\left(\partial r_{12}/\partial I_{2}\right)}^{2}s^{2}(I_{2})\\ \;=s^{2}(1+r_{12}^{2})/I_{2}^{2} \end{array}$$ (B1)

where

*r*_12_=*I*_1_*/I*_2_ and *s*^2^(*I*_1_)*=s*^2^. Thus, the errors in the intensity ratios are not normally distributed but instead, depend on the magnitude of *r* and the intensity in the ratio denominator. Correspondingly, each ratio is given a weighting factor in the least squares fit proportional to the inverse square of its uncertainty, namely,

$$W=I_{2}^{2}/(1+r_{12}^{2}).$$ (B2)

By setting the proportionality factor to unity rather than 1*/s*^2^ as suggested by eq (B1), the standard deviation returned by the fit should be a measure of the uncertainty of the Raman intensities. Comparison of the standard deviation of the fit with estimates of the uncertainty in the measured intensities demonstrated that eq (5) is capable of fitting the experimental data to within currently obtained measurement error.

The algorithm for the least squares analysis follows a Gram-Schmidt orthonormalization procedure. This procedure has been extensively tested [19] and a comparison between calculations using single and double precision gave identical results (to the degree of precision reported) indicating that the computation is free from numerical artifacts.

The major problem encountered in the analysis of the temperature profiles is associated with those situations in which the observed data are insufficient to determine the apparent phase transition temperature *T*_0_ and the scaling parameter *D.* The remainder of this section will deal with those situations.

The parameters *T*_0_ and *D* are determined solely from those data near *T* = *T*_0_, whereas *A, a, B* and *b* are determined primarily by data from the high and low temperature asymptotes of eq (5). The range around *T*_0_ for which *D* and *T*_0_ can be determined is the range in which *r* deviates significantly from the asymptotes *A+a*(*T−T*_0_) and *B+b*(*T−T*_0_). A deviation is defined as “significant” by the number of standard deviations by which a measured ratio differs from each of the asymptotes. In particular, for a measured ratio to contribute to the determination of *D* and *T*_0_, we require

$$\left|r_{i}-\left(A+a(T_{i}-T_{0})\right)\right|>fs/\surd W_{i}$$ (B3a)

and

$$\left|r_{i}-\left(B+b(T_{i}-T_{0})\right)\right|>fs/\surd W_{i}$$ (B3b)

where *r_i_* is the value of the intensity ratio at the observed temperature *T_i_ A, a, B, b* and *T*_0_ are the values of the parameters determined by the least squares fit; *W_i_* is the weight of the *i* th datum used in the least squares fit; *s* is the standard deviation returned by the least squares fit; and *f* is a multiplicative factor to allow for a more or less stringent test. The quantity, *s/√W_i_* is the estimated uncertainty in the experimental value of the *i*^th^ ratio. Equations (B3a,b) state that for the purpose of contributing to the evaluation of *T*_0_ and *D* the value of an observed ratio must differ from both asymptotic limits by an amount greater than some multiple *f* of the expected measurement uncertainty at that temperature. If the conditions of eqs (B3a,b) are met, we say that a measurement falls within the transition interval. Equations (B3a,b) define the temperature interval; the number of experimental data falling within this interval determines the reliability with which *D* and *T*_0_ can be estimated. In particular, the number of data falling within this interval is the appropriate number to use in calculating the number of degrees of freedom for the estimation of confidence intervals from a student’s *t* distribution.

If no measured temperature falls within the transition interval, neither *T*_0_ nor *D* can be estimated by a least squares fit of the data to eq (5). If one measured value falls within the interval, only *T*_0_ can be estimated, but not *D.* Furthermore, the value of the uncertainty in *T*_0_ returned by the least squares fit must be multiplied by a factor of 12.7, the value of *t* from the student’s *t* distribution for one degree of freedom and confidence limits of 95%. If two measured values fall within the interval, both *T*_0_ and *D* can be estimated, but the estimates of their uncertainties must be multiplied by 4.3, the value of *t* from the student’s *t* distribution appropriate for two degrees of freedom. Should more than two measured values fall within the transition interval, *T*_0_ and *D* can be determined with increasing reliability.

When less than two points fall within the transition interval, bounds can be placed on the values of *T*_0_ and *D*, even though *T*_0_ and *D* cannot be determined precisely. In order to place bounds on *T*_0_ and *D*, bounds must be placed on the transition interval. The transition interval can be estimated by combining eqs (B3a,b) with eq (5) to give, ignoring terms in *a* and *b*,

$$T_{x}=F_{x}D,$$

where

$$F_{x}=2\ln\left(\left|B-A\right|\frac{\surd W}{fs}-1\right)$$ (B4)

and *W* is the average weight of a measured value in the vicinity of the phase transition temperature. The transition interval *T_x_* is the temperature interval over which *r* varies from *A+a*(*T−T*_0_)*±fs/√W* to *B+b*(*T−T*_0_)∓*fs/√W*, where the upper sign is chosen if *A <B* and the lower sign if *B <A.*

If no measured temperature falls within the transition interval, as tested by eqs (B3a,b), we know that

$$0<T_{x}<T_{d}$$ (B5)

where *T_d_* is the temperature interval between the successive data pair above and below the phase transition temperature. Thus, from eq (B4)

$$0<D<T_{d}/F_{x}.$$ (B6)

In calculating the value of *A*, *a, B* and *b, D* is held fixed at its average value in the range defined by eq (B6); namely

$$D=T_{d}/2F_{x},$$ (B7)

and *T*_0_ is given as the midpoint between the two measured temperatures spanning the phase transition interval. Depending on the precision of the Raman intensity measurements relative to the difference between *A* and *B, F_x_* typically varies from 2 to 9. Most of the time a value of *f* = 2 is used in eqs (B3a,b) and (B4). Substitution of Δ*T* = 4*D* into eq (B6) yields

$$\Delta T=4RT_{0}^{2}/(\Delta \bar{H}n_{\text{eff}})<4T_{d}/F_{x}$$ (B8a)

and

$$n_{\text{eff}}>F_{x}RT_{0}^{2}/(\Delta \bar{H}T_{d}).$$ (B8b)

If one measured temperature *T_i_* falls within the transition interval,

$$0<T_{x}<2T_{d},$$ (B9)

where 2*T_d_ = T_i_+*1*−T_i_−*1 is twice the average data interval of the three measured temperatures spanning the transition interval. As with the case of no measured temperature falling within the interval, *D* is assigned the average value implied by the range of eq (B9); namely,

$$D=T_{d}/F_{x},$$ (B10)

and Δ*T* is reported as

$$\Delta T<8T_{d}/F_{x}.$$ (B11)

*T*_0_ is estimated by the least squares fit of all the data to eq (5) but is determined, almost exclusively, by the value of *r* at *T_i_* relative to *A* and *B.*
